# AP2/DREB Transcription Factor RAP2.4 Activates Cuticular Wax Biosynthesis in *Arabidopsis* Leaves Under Drought

**DOI:** 10.3389/fpls.2020.00895

**Published:** 2020-07-03

**Authors:** Sun Ui Yang, Hyojin Kim, Ryeo Jin Kim, Jungmook Kim, Mi Chung Suh

**Affiliations:** ^1^Department of Bioenergy Science and Technology, Chonnam National University, Gwangju, South Korea; ^2^Department of Life Science, Sogang University, Seoul, South Korea

**Keywords:** AP2/DREB-type, *Arabidopsis*, cuticular wax, drought, RAP2.4, transcription factor

## Abstract

Drought is a critical environmental stress that limits growth and development of plants and reduces crop productivity. The aerial part of land plants is covered with cuticular waxes to minimize water loss. To understand the regulatory mechanisms underlying cuticular wax biosynthesis in *Arabidopsis* under drought stress conditions, we characterized the role of an AP2/DREB type transcription factor, RAP2.4. *RAP2.4* expression was detected in one-week-old seedlings and rosette leaves, stems, stem epidermis, cauline leaves, buds, flowers, and siliques of 6-week-old *Arabidopsis*. The levels of *RAP2.4* transcripts increased with treatments of abscisic acid (ABA), mannitol, NaCl, and drought stress. Under drought, total wax loads decreased by approximately 11% and 10%, and in particular, the levels of alkanes, which are a major wax component, decreased by approximately 11% and 12% in *rap2.4-1* and *rap2.4-2* leaves, respectively, compared with wild type (WT) leaves. Moreover, the transcript levels of cuticular wax biosynthetic genes, *KCS2* and *CER1*, decreased by approximately 15–23% and 32–40% in *rap2.4-1* and *rap2.4-2* leaves, respectively, relative to WT 4 h after drought treatment, but increased by 2- to 12-fold and 3- to 70-fold, respectively, in three independent *RAP2.4* OX leaves relative to WT. Epicuticular wax crystals were observed on the leaves of *RAP2.4* OX plants, but not on the leaves of WT. Total wax loads increased by 1.5- to 3.3-fold in leaves of *RAP2.4* OX plants relative to WT. Cuticular transpiration and chlorophyll leaching occurred slowly in the leaves of *RAP2.4* OX plants relative to WT. Transcriptional activation assay in tobacco protoplasts showed that RAP2.4 activates the expression of *KCS2* and *CER1* through the involvement of the consensus CCGAC or GCC motifs present in the *KCS2* and *CER1* promoter regions. Overall, our results revealed that RAP2.4 is a transcription factor that activates cuticular wax biosynthesis in *Arabidopsis* leaves under drought stress conditions.

## Introduction

Drought is a severe environmental stress implicated in the reduction of plant growth and crop productivity. As plants are sessile, they must cope with drought stress for their optimal growth and development. During the transition of land plants from aquatic to terrestrial environments, they developed a key surface structure, the outermost cuticle layer covering their aerial tissues, to protect them from terrestrial stresses such as drought, excess light, and UV light (Shepherd and Griffiths, 2006; Bernard and Joubès, 2013; Yeats and Rose, 2013). Therefore, the primary function of the cuticle layer is to reduce water-loss through the epidermis, except through the stomata (Yeats and Rose, 2013; Lee and Suh, 2015a). The cuticle, which is the first physical barrier between plants and the environment, plays a role in the protection of plants from excess UV light, pathogenic spores, and insect attack (Eigenbrode and Espelie, 1995; Sieber et al., 2000; Holmes and Keiller, 2002; Yeats and Rose, 2013) and also in the prevention of organ fusion during plant development (Ingram and Nawrath, 2017). The hydrophobic cuticle layer mainly comprises the cutin polyester matrix, intracuticular waxes embedded in the cutin matrix, and epicuticular waxes. Cutin polyester is mainly formed by interlinking through ester bonds of ω-hydroxy C16 or C18 fatty acids and their derivatives (Beisson et al., 2012; Bakan and Marion, 2017). Major components of cuticular waxes are very long chain fatty acids (VLCFA, longer than C20) and their derivatives including alkanes, aldehydes, primary and secondary alcohols, and wax esters (Lee and Suh, 2013; Hegebarth and Jetter, 2017).

Cuticular wax biosynthesis mainly occurs in the epidermal cells of plants (Suh et al., 2005). The C16 and C18 fatty acids synthesized in the plastids are further elongated to VLCFAs by the fatty acid elongase (FAE) complex in the endoplasmic reticulum (ER) (Millar et al., 1999; Zheng et al., 2005; Bach et al., 2008; Beaudoin et al., 2009; Kunst and Samuels, 2009; Haslam and Kunst, 2013; Kim et al., 2013; Morineau et al., 2016). The VLCFAs are modified to VLC aliphatic compounds, which are aldehydes, alkenes, secondary alcohols, and ketones via the alkane-forming pathway and primary alcohols and wax-esters via the alcohol-forming pathway (Rowland et al., 2006; Greer et al., 2007; Li et al., 2008; Bourdenx et al., 2011; Li-Beisson et al., 2013; Bernard and Joubès, 2013; Lee and Suh, 2015a). The wax molecules synthesized in the ER are secreted through the ATP-binding cassette transporter in the plasma membrane and with the help of glycosylphosphatidyl-anchored lipid transfer proteins to extracellular space, and then deposited on the surface of epidermal cells (DeBono et al., 2009; Lee et al., 2009; Kim et al., 2012). The total wax load and composition varied greatly among plant species, in an organ-specific manner, or by environmental factors (Holmes and Keiller, 2002; Shepherd and Griffiths, 2006; Kosma et al., 2009; Seo et al., 2011; Go et al., 2014; Lee et al., 2016; Kim et al., 2018). In particular, the total wax load increased by approximately 2-fold in *Arabidopsis* and tree tobacco (*Nicotiana glauca*) leaves under drought stress conditions (Kosma et al., 2009; Seo et al., 2011; Lee et al., 2016). Increased total wax loads in leaves reduced cuticular transpiration rate and conferred resistance to drought (Seo et al., 2011; Lee et al., 2016). Noticeably, the increased levels of VLC-alkane components in *Arabidopsis* are prominent under drought stress conditions, indicating that the molecular regulatory mechanism underlying the alkane-forming pathway may be important to understand one of the plant strategies to cope with drought (Kosma et al., 2009; Xue et al., 2017).

It has long been questioned how land plants precisely regulate total wax loads in response to drought stress. Several transcription factors have been reported to be implicated in the upregulation of wax biosynthesis in plants under drought (Seo et al., 2011; Lee et al., 2016). WIN1/SHN1, which belongs to the AP2/ERF transcription factor family, is a transcriptional activator that induces the expression of *KCS1*, *ECERIFERUM1* (*CER1)*, and *CER2* genes and its overexpression in *Arabidopsis* and *N. tabacum* caused increased resistance to drought (Aharoni et al., 2004; Broun et al., 2004; Kannangara et al., 2007; Djemal and Khoudi, 2019). Similarly, ectopic expression of *Medicago truncatula WXP1* and *WXP2* transcription factors containing the AP2 domain led to increased wax load and enhanced drought tolerance (Zhang et al., 2005; Zhang et al., 2007). In addition, the *Arabidopsis* R2R3-type transcription factor MYB96, which is induced by drought and ABA treatment, directly activates the expression of *KCS1, KCS2, KCS6, KCR1*, and *CER3* genes involved in wax biosynthesis (Seo et al., 2011). Its functional homolog, MYB94, is a transcription activator that increases the levels of *WSD1, KCS2, CER2, FAR3*, and *ECR* transcripts in *Arabidopsis* (Lee and Suh, 2015b). MYB94 and MYB96 additively activate cuticular wax biosynthesis by binding to the same MYB consensus motifs in wax biosynthetic gene promoters (Lee et al., 2016). Interestingly, total wax loads of double knockout *myb94 myb96* leaves decreased by approximately 44% and 52% relative to the wild type under well-watered and drought stress conditions, respectively, indicating that about 50% of total wax biosynthesis in *Arabidopsis* leaves is dependent on both MYB94 and MYB96 (Lee et al., 2016). However, it is still unclear how the biosynthesis of the remaining wax in *myb94 myb96* is regulated in *Arabidopsis* under both conditions.

*Arabidopsis* transcriptome analysis showed that the expression of an AP2/DREB transcription factor, RELATED TO APETALA 2.4 (RAP2.4, At1g78080) was induced by drought and salt stress treatments (Feng et al., 2005). *M. truncatula* WXP1, which shares the highest sequence homology with *Arabidopsis* RAP2.4, displayed increased total wax loads and enhanced drought tolerance in transgenic alfalfa (*Medicago sativa*) (Zhang et al., 2005). Lin et al. (2008) observed enhanced drought tolerance in *Arabidopsis* overexpressing *RAP2.4* (*RAP2.4* OX), but no significant differences were detected in water loss between wild type and *RAP2.4* OX lines. These results suggest that regulation of cuticular wax biosynthesis may be involved in the drought stress response mediated by *Arabidopsis* RAP2.4.

In this study, we investigated the role of an AP2/DREB-type transcription factor, RAP2.4 in cuticular wax biosynthesis in *Arabidopsis* leaves under drought stress condition. Quantitative Real-Time-PCR (qRT-PCR) analysis showed that *RAP2.4* expression is induced by treatment with ABA, and drought, salt, and osmotic stress. Under drought stress condition, total wax loads significantly decreased in *Arabidopsis rap2.4-1* and *rap2.4-2* leaves relative to the wild type under drought stress conditions, but no remarkable differences between the wild type and *rap2.4* mutants were observed under well-watered conditions. In addition, we observed that ectopic expression of *RAP2.4* increased total wax loads in *Arabidopsis* leaves and delayed water-loss through the cuticular layer of leaves. Transactivation assay of RAP2.4 in *N. benthamiana* protoplasts revealed that the expression of *KCS2* and *CER1* involved in VLCFA and alkane biosynthesis, respectively, is induced by *RAP2.4* expression and in particular, the CCGAC or GCC consensus motifs present in the *KCS2* and *CER1* promoter regions are required for RAP2.4-mediated elevation of *KCS2* and *CER1* expression. These results demonstrate that an AP2/DREB-type transcription factor, RAP2.4 activates cuticular wax biosynthesis by increasing the expression of *KCS2* and *CER1* in *Arabidopsis* leaves under drought. This study suggests that RAP2.4, in addition to MYB96 and MYB94, is also an important component of the transcriptional gene regulatory network involved in drought-induced wax biosynthesis in *Arabidopsis*.

## Materials and Methods

### Plant Materials and Growth Conditions

The *Arabidopsis* T-DNA insertion mutants *rap2.4-1* (SALK_020767) and *rap2.4-2* (SALK_093377) were obtained from the Arabidopsis Biological Resource Center (ABRC^1^). To isolate *rap2.4* mutants, the genomic DNA was isolated from rosette leaves of 2-week-old *Arabidopsis* using DNA extraction buffer [200 mM Tris-Cl (pH 8.0), 250 mM NaCl, 25 mM EDTA (pH 8.0), 0.5% SDS]. Genomic DNA and gene-specific primers (Supplementary Table 1) were used for DNA-based PCR. The seeds of *Arabidopsis* wild type (Col-0), transgenic *Arabidopsis* lines overexpressing *RAP2.4*, and *rap2.4 Arabidopsis* mutants were surface-sterilized with 75% EtOH solution containing 0.05% Triton X-100 and 100% EtOH. The *Arabidopsis* seeds were germinated in 1/2 MS medium (0.22% Murashige and Skoog media, 1% sucrose, 0.7% phytoagar, pH 5.7). Transgenic *Arabidopsis* seeds were germinated in 1/2 MS medium containing kanamycin (25 μg/ml). Seven-day-old *Arabidopsis* seedlings were transferred to sterile soil (soil:vermiculite:perlite, 3:2:1). All growth conditions were maintained at 24 ± 2°C under long-day conditions (16 h/8 h).

For treatment of drought stress, 7-day-old seedlings of wild type (Col-0) and *rap2.4* mutants were transferred to plastic pots [350 (W) × 270 (D) × 130 (H)] filled with 500 g of soil. The transferred seedlings were covered with plastic wrap to maintain humidity for 5 days, The plastic wrap was punched with a razor blade to maintain ambient humidity; the plastic wrap was removed after 2 days. The soil was first soaked with water and then the plants were exposed to drought stress for 2 weeks. Next, the soil was re-soaked with water and then, the plants were subjected to a second drought treatment for 2 weeks.

### Construction of Binary Vectors and *Arabidopsis* Transformation

To generate transgenic *Arabidopsis* overexpressing *RAP2.4*, *RAP2.4* cDNA was amplified using At1g78080 F1/At1g78080 R1 primers (Supplementary Table 1) and *Arabidopsis* seedling cDNA. The amplified products were translationally fused with the gene encoding enhanced yellow fluorescent protein (eYFP) between the CaMV35S promoter and the terminator of ribulose 1,5-biphosphate carboxylase/oxygenase small subunit (rbc-T) from *Pisum sativum* in the modified pPZP212 binary vector^2^ (Hajdukiewicz et al., 1994). The recombinant vector was introduced into *Arabidopsis* (ecotype, Col-0) using *Agrobacterium*-mediated transformation (Clough and Bent, 1998). Transgenic *Arabidopsis* seedlings (*RAP2.4* OX) were selected in 1/2 MS medium containing carbenicillin (100 μg/ml) and kanamycin (25 μg/ml). The selected transgenic plants (T_2_ or T_3_ plants) were used for further experiments.

### Isolation of Total RNA and Gene Expression Analysis

To analyze the expression of *RAP2.4* in various organs or tissues of *Arabidopsis*, we collected 1-week-old seedlings and rosette leaves, stems, flowers, buds, and siliques of 6-week-old *Arabidopsis*. To investigate the expression patterns after treatment of osmotic stress, salt stress, or exogenous ABA, 10-day-old seedlings were floated and incubated in 1/2 MS liquid medium containing 200 mM mannitol, 200 mM NaCl, or 100 μM ABA for 1, 2, and 6 h at 25]°C with shaking at 40 rpm. For exposure to drought stress, 10-day-old seedlings were transferred to cellulose paper (Whatman) and air-dried for 1 and 2 h. To investigate the expression patterns of cuticular wax biosynthetic genes in 4-week-old *Arabidopsis* wild type and *rap2.4* mutants after the drying event, the aerial parts of wild type and *rap2.4* mutants were cut and placed on the cellulose paper and air-dried for 1, 2, and 4 h. To analyze the expression patterns of cuticular wax biosynthetic genes in the wild type and transgenic *Arabidopsis* overexpressing *RAP2.4*, the rosette leaves were collected from 4-week-old *Arabidopsis*. Total RNA was isolated using the Nucleospin RNA Plant Extraction Kit (MACHEREY-NAGEL) following the manufacturer’s protocols. Total RNA was used for cDNA synthesis using Gostrip^TM^ Reverse Transcriptase (Promega) following the manufacturer’s protocols. The cDNA and gene-specific primers (Supplementary Table 1) were used for both the semi-RT-PCR and the quantitative RT-PCR (CFX96 thermal cycler, Bio-Rad). The KAPA SYBR FAST qRT-PCR kit (KAPA Biosystems) was used for quantitative RT-PCR. Quantification of the *PP2AA3* (At1g13320) transcripts was used for normalization of the *RAP2.4* transcripts and cuticular wax biosynthetic genes.

### Scanning Electron Microscope (SEM) Analysis

To observe the cuticular wax crystal on the surface of leaves, rosette leaves from 4-week-old wild type and transgenic *Arabidopsis* overexpressing *RAP2.4* were used according to methods described in a previous study (Lee and Suh, 2015b).

### Cuticular Wax Analysis Using Gas Chromatography (GC)

The cuticular wax were extracted from rosette leaves of 5- to 6-week-old wild type, *rap2.4* mutants, and three independent transgenic *Arabidopsis* overexpressing *RAP2.4* (OX20, OX26, and OX31) by shaking in chloroform for 30 s. Extracts were evaporated under nitrogen gas after adding the internal standard (1-tricosanol, docosanoic acid, and *n*-octacosane). Then, concentrated wax was dissolved in a solution of pyridine and bis-*N,O*-trimethylsilyl trifluoroacetamide (BSTFA, Sigma) (1:1, v/v) and incubated at 90°C for 30 min. The silylated waxes were concentrated under nitrogen gas and they were re-dissolved with premixed heptane-toluene solution (1:1, v/v). The conditions of the GC analysis were described in a previous study (Lee and Suh, 2015b).

### Transpiration and Chlorophyll Leaching Assays

For the cuticular transpiration assay, well-watered 3-week-old *Arabidopsis* plants were incubated in the dark for 12 h, and the shoot was carefully separated from the root. Subsequently the shoot was fully watered for 1 h by floating on water. After removal of water from the shoot surface, the weights of the shoots were measured for 2.5 h with 15 min. intervals while maintaining the dark condition.

For the chlorophyll leaching assay, the shoots without roots of well-watered 3-week-old *Arabidopsis* were exposed to the dark for 12 h, and then immediately immersed in 80% ethanol with shaking at 40 rpm. The extracted chlorophyll content was measured for 3 h with 15 min. intervals on a spectrophotometer (Ultrasec 3100 pro; Amersham Biosciences) at wavelengths of 647 and 664 nm. The chlorophyll content was calculated as μmol Chlorophyll/fresh weight (FW, g) = [7.93 (A664) + 19.53 × (A647)]/FW (g).

### Transcriptional Activation Assay in *N. benthamiana* Protoplasts

The transcriptional activation assays were performed as described in Lee and Suh (2015b). For making reporter constructs, the minimal *CaMV 35S* promoter and *LUC* gene of *Gal4(3X):LUC* plasmid (Tiwari et al., 2003) were amplified with Min35S pro_*Pst*I-F/Min35S pro_*Xba*I-R primer set and Luc *Xma*I F/Luc *Sac*I R primer set, respectively, and then the *CaMV 35S* promoter and *GUS* gene of pBI221 were replaced with the amplified DNA fragments, minimal *CaMV 35S* promoter and *LUC* gene, respectively (Supplementary Table 1). The generated vector named as pBI:LUC. Next, the oligonucleotides (KCS2 BS1, KCS2 BS2, CER1 BS1, and CER1 BS2) containing the consensus CCGAC or GCC motifs, which are present in the promoter regions of *KCS2* and *CER1* and their mutated oligonucleotides (KCS2 mBS1, KCS2 mBS2, CER1 mBS1, and CER1 mBS2), where the consensus CCGAC and GCC sequences were changed to TTTTT and TTT sequences, were designed. The *Hin*dIII and *Sal*I restriction enzyme sites were included in the designed oligonucleotides at 5’- and 3’-end, respectively. The synthesized oligonucleotides were annealed, and then the DNA fragments were cloned in *Hin*dIII and *Sal*I sites of the *pBI:LUC* vector. The *pPZP212* and *pRAP2.4* binary vectors were used as the effector. The *pPZP212* or *pRAP2.4.* binary vector was co-transfected with each reporter vector into *N. benthamiana* leaf protoplast using polyethylene glycol (PEG)-mediated transfection (Yoo et al., 2007). The *pBI221* expression vector was used as the normalization factor. Twenty hours after transfection, total protein extracted from the protoplasts was subjected to enzymatic assays to quantifyluciferase (LUC) activity using a dual-luciferase assay system (Promega). Quantification of the GUS level was used for normalization of quantification of LUC activity. The fluorescence of LUC and GUS were detected under GLOMAX (Promega).

## Results

### Expression of *RAP2.4* in *Arabidopsis*

**FIGURE 1:**
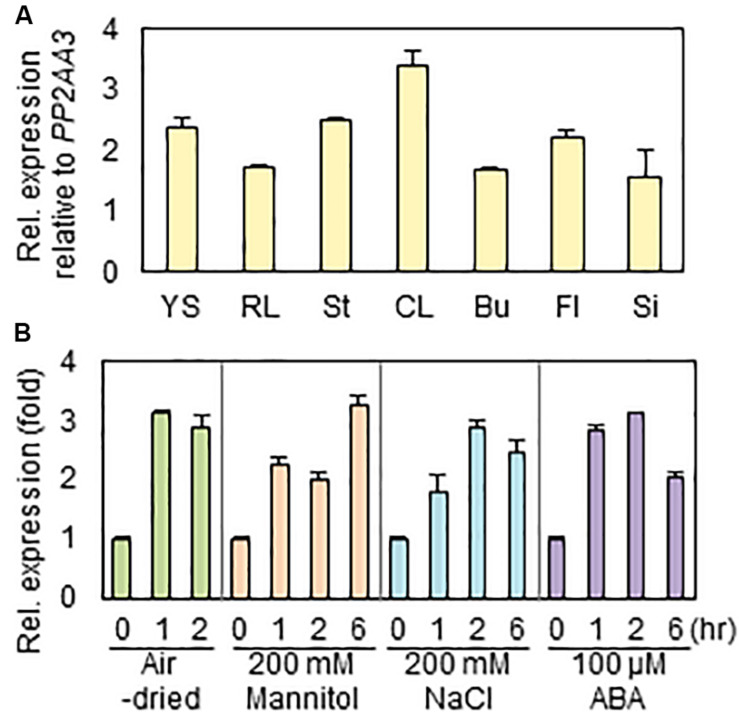
Expression of *RAP2.4* in *Arabidopsis*. **(A)** The transcript level of *RAP2.4* in various organs of *Arabidopsis*. Total RNA was isolated from 1-week-old seedlings (YS), rosette leaves (RL), stems (St), cauline leaves (CL), buds (Bu), flowers (Fl), and silique (Si) of 6-week-old *Arabidopsis*. Transcript levels were determined by quantitative Real Time-Polymerase Chain Reaction (qRT-PCR) and relative expression was determined using the ΔΔCt method. *PP2AA3* (At1g13320) was used to determine the quantity and quality of the cDNAs. Each value is the mean ± SD of three independent measurements. **(B)** Expression of *RAP2.4* transcripts after application of exogenous ABA and treatment of salt and osmotic stress, and drought. One-week-old seedlings were transferred to the 1/2 MS liquid medium supplemented with 200 mM mannitol, 200 mM NaCl, or 100 μM ABA for the indicated times. For drought stress, the transferred seedlings were air-dried for the indicated times. Transcript levels were determined by qRT-PCR and relative expression was determined using the ΔΔCt method. *PP2AA3* (At1g13320) was used to determine the quantity and quality of the cDNAs. Each value is the mean ± SD of three independent measurements.

To investigate the expression levels of *RAP2.4* gene in various *Arabidopsis* organs, we isolated total RNAs from 7-day-old seedlings and rosette leaves, stem, cauline leaves, floral buds, flowers, and siliques of 6-week-old plants, and then subjected them to qRT-PCR. When the transcript level of *RAP2.4* was compared with that of *PP2AA3*, which is used as a reference gene to assess the quantity and quality of RNA samples (Czechowski et al., 2005), the level of *RAP2.4* transcripts was approximately 1.6- to 3.4-fold higher in the various *Arabidopsis* organs tested (Figure 1A). The highest expression of *RAP2.4* was observed in cauline leaves. To measure the increased levels of *RAP2.4* transcripts in *Arabidopsis* after drought, osmotic, and salt stress treatments or ABA applications, 10-day-old seedlings were air-dried or incubated on 1/2 MS media supplemented with 200 mM mannitol, 200 mM NaCl, or 100 μM ABA for 0, 1, 2, or 6 h. The transcript levels of *RAP2.4* increased by approximately 2.9- to 3.1-fold with drought treatment, 2.0- to 3.2-fold with 200 mM mannitol treatment, 1.8- to 2.9-fold with salt stress, and 2.1- to 3.1-fold with ABA applications (Figure 1B).

### Isolation of *rap2.4* Knock-Out Mutants and Generation of Transgenic Arabidopsis Plants Overexpressing *rap2.4*

**FIGURE 2:**
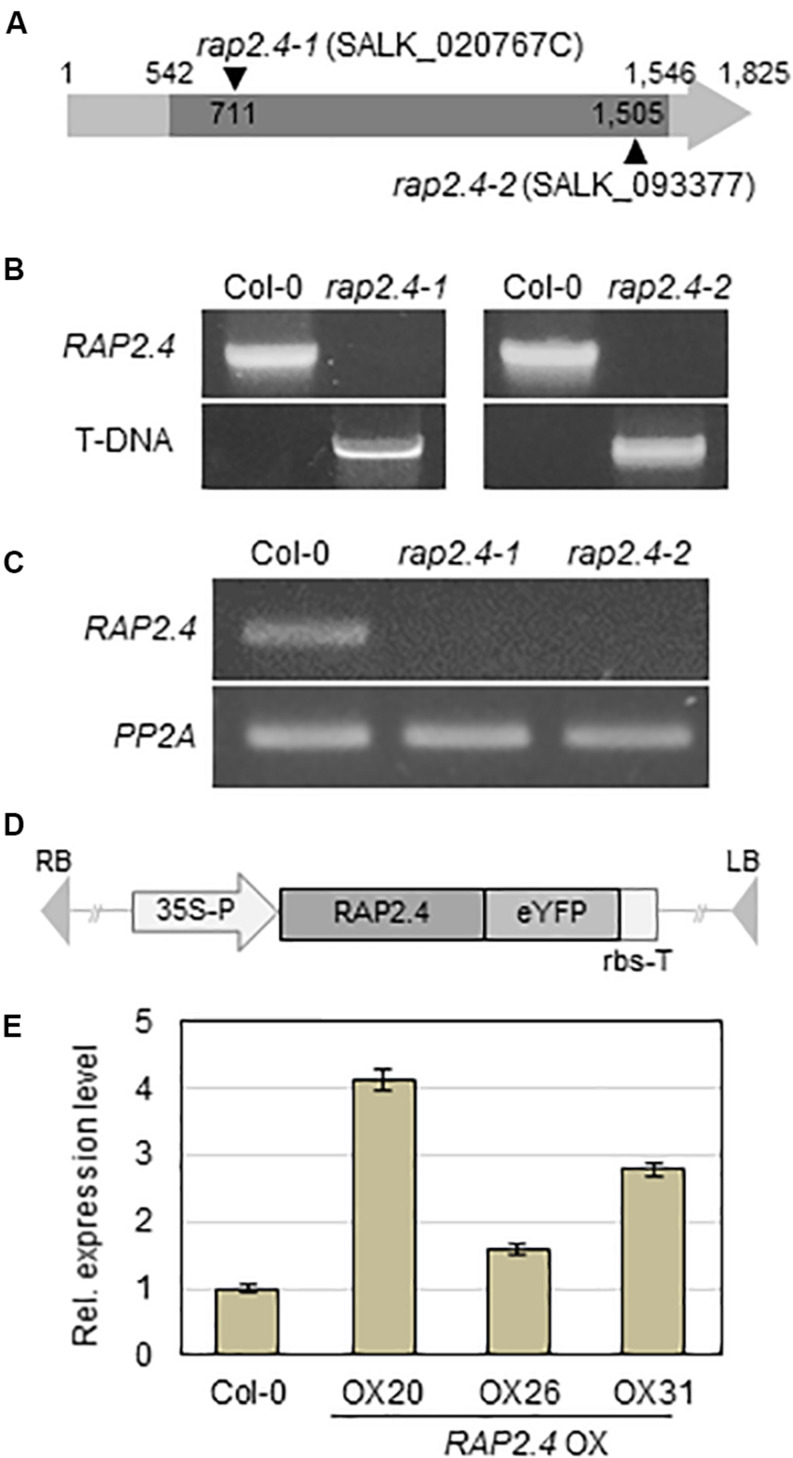
Isolation of *Arabidopsis RAP2.4* knock-out mutant and generation of transgenic *Arabidopsis* overexpressing *RAP2.4*. **(A)** Schematic diagram of T-DNA-inserted *Arabidopsis rap2.4* mutants. The *rap2.4-1* and *rap2.4-2* are *RAP2.4* knock-out mutant alleles. Genomic DNA-PCR **(B)** and reverse transcription (RT)-PCR **(C)** analyses of *RAP2.4* in *Arabidopsis* wild type (Col-0) and *rap2.4* mutants. Total RNA was extracted from 4-week-old rosette leaves and subjected to RT-PCR. *PP2AA3* (At1g13320) was used to determine the quantity and quality of the cDNAs. **(D)** Schematic diagram of *RAP2.4* overexpression construct under the control of the CaMV 35S promoter (35S-P). rbs-T, terminator of ribulose 1,5-biphosphate carboxylase/oxygenase small subunit from *Pisum sativum*. eYFP, enhanced yellow fluorescent protein. RB, right border. LB, left border. **(E)** Quantitative real-time (RT)-PCR analysis of three independent transgenic *Arabidopsis* plants overexpressing *RAP2.4* (OX20, OX26, and OX31). Total RNA was extracted from 4-week-old rosette leaves. *PP2AA3* (At1g13320) was used to determine the quantity and quality of the cDNAs. Transcript levels were determined by qRT-PCR and relative expression was determined using the ΔΔCt method. Each value is the mean of three independent measurements. Bars indicate standard deviation.

To investigate the role of the *RAP2.4* gene in cuticular wax biosynthesis, T-DNA-inserted *rap2.4* mutant seeds (SALK_020767C and SALK_093377) were obtained from the Arabidopsis Biological Resource Center (www.arabidopsis.org). The T-DNA insertions were designated between 710 and 711 bp from initiation of transcription in *rap2.4-1* (SALK_020767C) and 1504 and 1505 bp from initiation of transcription in *rap2.4-2* (SALK_093377) in the TAIR site (see footnote 1) (Figure 2A). To check the T-DNA insertions from *rap2.4-1* and *rap2.4-2* mutants, genomic DNA was extracted from leaves of *rap2.4-1* and *rap2.4-2*, and then genomic DNA PCR was performed. PCR bands, which were amplified using At1g78080-NF1 and At1g78080-CR1 primers, were detected in wild type (Col-0) but not in *rap2.4-1* and *rap2.4-2* mutants. However, PCR bands, which were amplified using LBa1 and At1g78080-CR1 or At1g78080-NF1 and LBa1 primers, were detected in *rap2.4-1* and *rap2.4-2* mutants but not in the wild type (Col-0) (Figure 2B). In RT-PCR analysis of 4-week-old wild type *rap2.4-1*, and *rap2.4-2* leaves, *RAP2.4* transcripts were detected in the wild type but not in *rap2.4-1* and *rap2.4-2*, indicating that *rap2.4-1* and *rap2.4-2* are knockout mutants (Figure 2C).

To generate transgenic *Arabidopsis* plants overexpressing *RAP2.4*, *RAP2.4* was inserted between the *CaMV 35S* promoter and the terminator of ribulose 1,5-biphosphate carboxylase/oxygenase small subunit (rbs-T) (Figure 2D). The generated binary vector was transformed into *Arabidopsis* plants via the *Agrobacterium*-mediated transformation method. qRT-PCR showed that the levels of *RAP2.4* transcripts in *RAP2.4* overexpression lines, OX20, OX26, and OX31 increased by 4.7-fold, 1.6-fold, and 2.8-fold, respectively, compared to the wild type (Figure 2E). During growth and development, we observed that growth of *RAP2.4* OX lines was smaller than that of the wild type and the growth-retarded phenotype was prominent in *RAP2.4* OX20. However, no noticeable differences in growth and development were observed between wild type and *rap2.4* mutants (Supplementary Figure 1).

### Cuticular Wax Content and Composition in Arabidopsis Wild Type and *rap2.4* Mutants Under Well-Watered and Drought Stress Conditions

**FIGURE 3:**
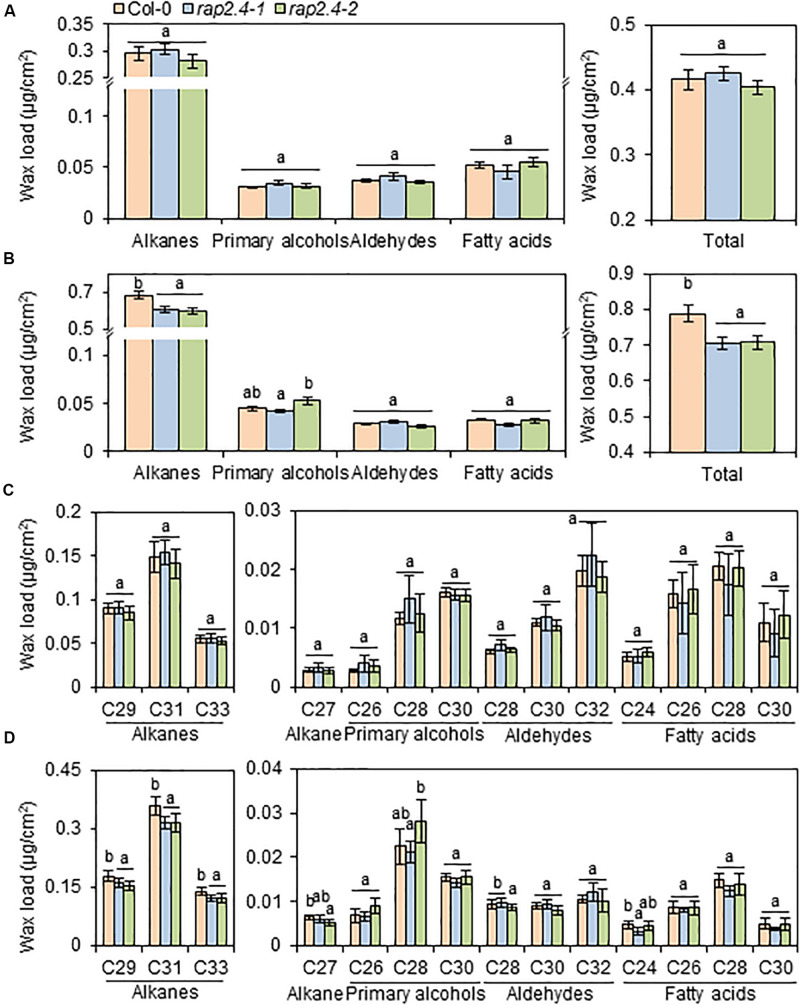
Cuticular wax amount and composition in the leaves of *Arabidopsis* wild type (Col-0) and *rap2.4* mutants under normal and drought conditions. **(A)** Cuticular wax amount in leaves of plants grown under the normal growth conditions (temperature, 24 ± 2°C; humidity, approximately 50%; light condition, 100–120 μmol m^–2^s^–1^). **(B)** Cuticular wax amount in leaves of plants grown under the drought stress conditions. **(C)** Cuticular wax composition in leaves of plants grown under normal growth conditions. **(D)** Cuticular wax composition in leaves of plants grown under drought stress conditions. Cuticular waxes were extracted from leaves of 5- to 6-week-old *Arabidopsis* grown under normal growth and drought stress conditions. Each value is the mean of three independent measurements. Bars indicate standard deviation. Different letters denote statistically significant differences at *P* < 0.05 (Tukey’s test), following a one-way ANOVA test with treatment as the variable factor.

To investigate the effect of loss-of-function of *RAP2.4* on cuticular wax composition and amount, cuticular waxes were extracted from leaves and stems of wild type, *rap2.4-1*, and *rap2.4-2* mutants which were grown under well-watered or drought stress conditions, and measured by gas chromatography (GC) with a flame ionization detector (FID). Wild type and *rap2.4* mutants showed no significant differences in leaf wax content and composition under sufficiently watered conditions (Figures 3A,C). Under the drought stress condition, however, total wax loads decreased by approximately 10% in both *rap2.4-1* and *rap2.4-2* leaves relative to the wild type (Figures 3B,D). Approximately 13–19%, 11–14%, 11–12%, and 12% reduction in the levels of each VLC-alkane, C27, C29, C31, and C33, respectively, were observed in *rap2.4* mutants relative to the wild type under drought (Figures 3B,D). However, there were no significant differences in the levels of other wax components even though plants were grown under drought (Figures 3B,D).

### Epicuticular Wax Crystals and Cuticular Wax Content and Composition in *Arabidopsis* Wild Type and *RAP2.4* Overexpression Lines

**FIGURE 4:**
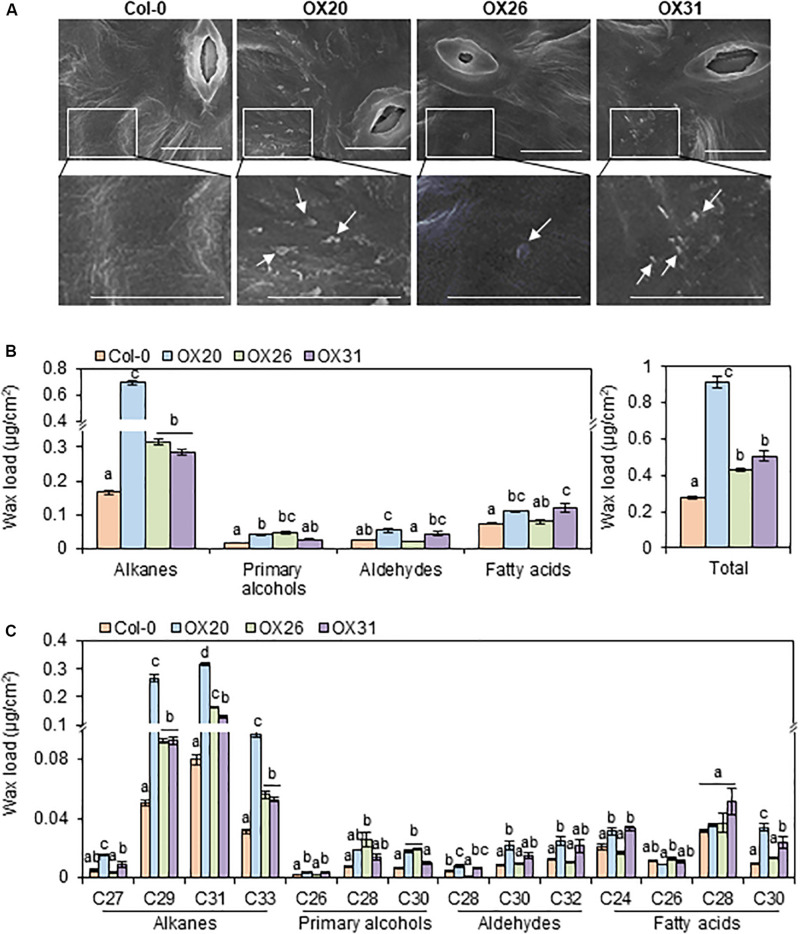
Epicuticular wax crystals **(A)** and cuticular wax amount and composition **(B)** in the leaves of *Arabidopsis* wild type (Col-0) and *RAP2.4* OX lines. **(A)** Scanning electron microscopy analysis of epicuticular wax crystals on leaves of 3- to 4-week-old *Arabidopsis* wild type (Col-0) and *RAP2.4* OX lines. The images (inset) in the upper panel were magnified and shown in the lower panel. Bars = 10 μm. White arrows indicate wax crystals. **(B)** Cuticular wax amount in leaves of 5-week-old *Arabidopsis* wild type (Col-0) and *RAP2.4* OX lines. **(C)** Cuticular wax composition in leaves of 5-week-old *Arabidopsis* wild type (Col-0) and *RAP2.4* OX lines. Each value is the mean of three independent measurements. Bars indicate standard error. Different letters denote statistically significant differences at *P* < 0.05 (Tukey’s test), following a one-way ANOVA test with treatment as the variable factor.

Because the wax-deficient phenotype of *rap2.4* mutants was observed under drought, we next examined the formation of epicuticular wax crystals in leaves of wild type and *RAP2.4* overexpression lines using SEM. SEM analysis showed that epicuticular wax crystals were clearly observed in leaves of *RAP2.4* OX20, OX26, and OX31 lines, but not in leaves of the wild type (Figure 4A).

Subsequently, the content and composition of cuticular waxes were measured from leaves and stems of wild type and *RAP2.4* overexpression lines using GC-FID (Supplementary Figure 2). Total wax content increased by approximately 3.3-, 1.5-, and 1.8-fold in *RAP2.4* OX20, OX26, and OX31, respectively, compared with to that in the wild type (Figure 4B). The largest increase (1.7- to 4.2-fold) in leaves of all three *RAP2.4* OX lines relative to that in the wild type was observed in the levels of VLC-alkanes (C27, C29, C31, and C33), which are major wax components (more than 70% of total wax loads) in *Arabidopsis* leaves (Figure 4C). In particular, approximately 3.2-, 5.3-, 4.0-, and 3.1-fold increase in the levels of C27, C29, C31, and C33, respectively, was observed in leaves of *RAP2.4* OX20 compared with that of the wild type (Figure 4C). The significant increase in the levels of C29, C31, and C33 VLC-alkanes was also observed in leaves of *RAP2.4* OX26, and OX31 relative to wild type (Figure 4C). An increase in the levels of other components, VLC-primary alcohols (C26, and C28), VLC-aldehydes (C28, C30, and C32), and VLC-fatty acids (C24 and C30) was also detected in leaves of *RAP2.4* OX20 and OX31 relative to the levels in the wild type, except for C30 VLC-PA and C28 VLCFAs (Figure 4C). The levels of C28 and C30 VLC-PA were increased in leaves of *RAP2.4* OX26 relative to wild type (Figure 4C).

### Cuticular Transpiration and Chlorophyll Leaching Assays in *Arabidopsis* Wild Type and *RAP2.4* Overexpression Lines

**FIGURE 5:**
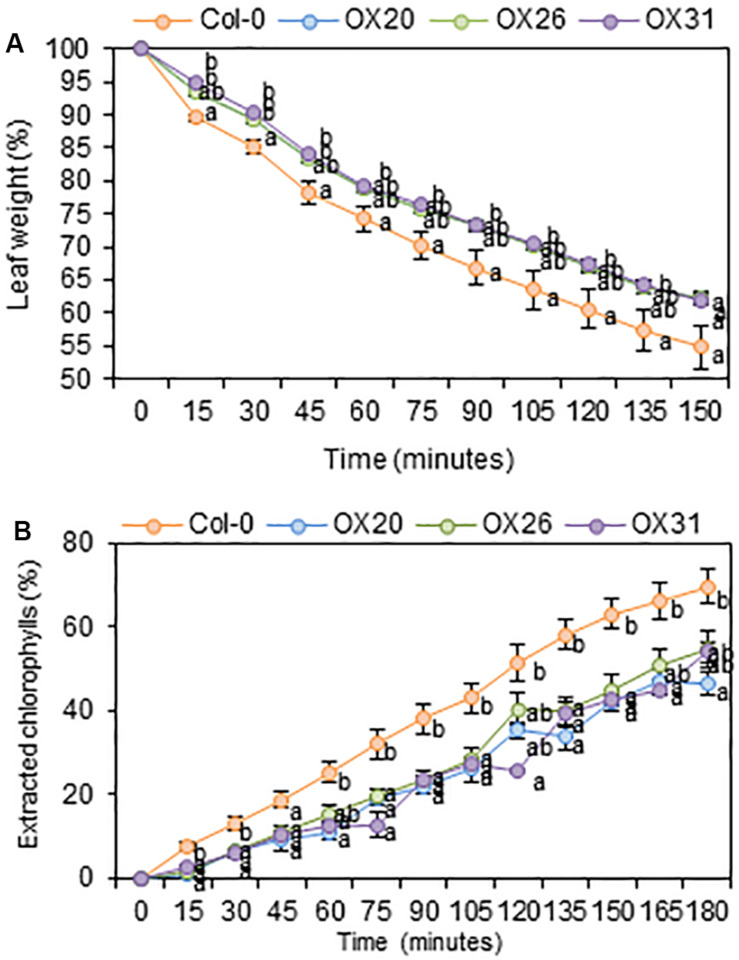
Cuticular transpiration **(A)** and chlorophyll leaching **(B)** assays of *Arabidopsis* wild type (Col-0) and *RAP2.4* OX lines. **(A)** Cuticular transpiration assay in leaves of 3-week-old *Arabidopsis* wild type (Col-0) and *RAP2.4* OX lines. **(B)** Chlorophyll leaching in leaves of 3-week-old *Arabidopsis* wild type (Col-0) and *RAP2.4* OX lines. Each value represents the mean of three independent measurements. Bars indicate the standard error of the mean. Different letters denote statistically significant differences at *P* < 0.05 (Tukey’s test), following a one-way ANOVA test with treatment as the variable factor.

An increase in total wax loads in leaves of *RAP2.4* OX lines prompted us to measure cuticular transpiration and chlorophyll leaching assays in the *Arabidopsis* wild type and *RAP2.4* overexpression lines. For the cuticular transpiration assay, 3-week-old wild type and *RAP2.4* OX lines were dark-treated for 12 h to completely close stomata in leaves, whole aerial parts of plants were floated on water under dark for 1 h, and then, cuticular transpiration rate was measured. As shown in Figure 5A, the water loss through the cuticle was slower in all three *RAP2.4* OX lines than in the wild type.

For the chlorophyll leaching assay, 3-week-old wild type and *RAP2.4* OX lines were incubated for 12 h under dark, whole aerial parts of the plants were immersed in 80% ethanol, and then, the chlorophyll content leached from the leaves was measured. As shown in Figure 5B, the rate of chlorophyll leaching was slower in the *RAP2.4* OX lines than in the wild type.

### Expression of Genes Involved in Cuticular Wax Biosynthesis and Its Regulation in Arabidopsis Wild Type, *rap2.4* Mutants, and *RAP2.4* OX Leaves Under Well-Watered and/or Drought

**FIGURE 6:**
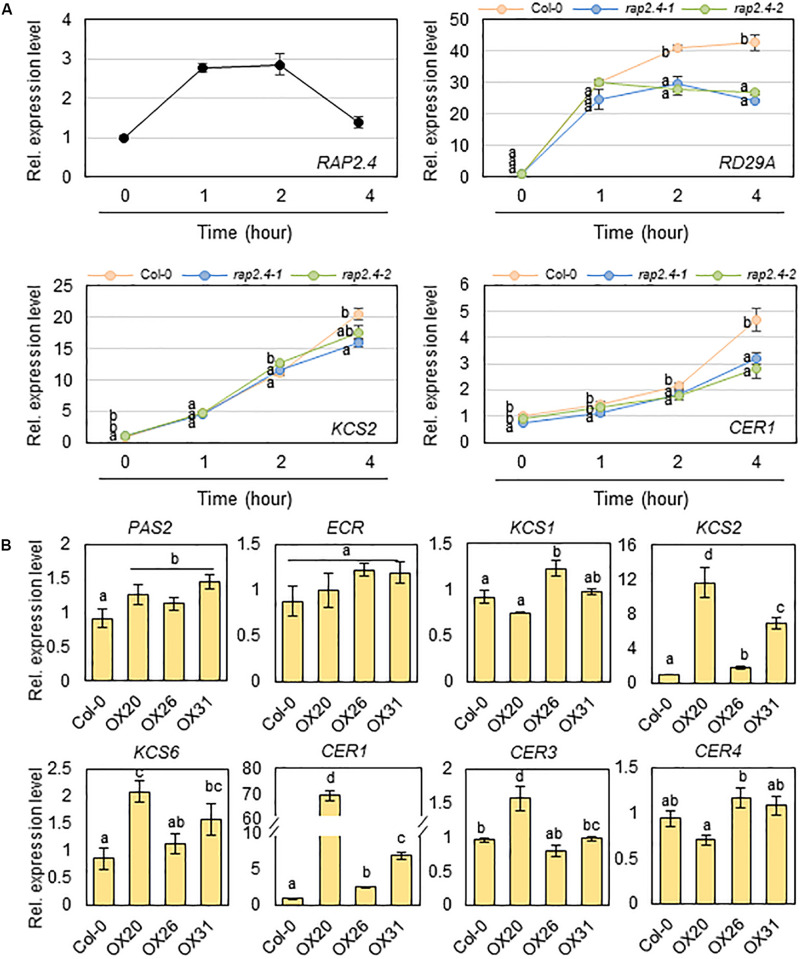
Quantitative RT-PCR analysis of wax biosynthetic genes in 4-week-old wild type (Col-0), *rap2.4* mutants, and *RAP2.4* OX lines. **(A)** The transcript level of *RAP2.4* in 4-week-old *Arabidopsis* wild type (Col-0) and the transcript level of *RD29A*, *KCS2*, and *CER1* in 4-week-old *Arabidopsis* wild type (Col-0) and *rap2.4* mutants. Rosette leaves from 4-week-old *Arabidopsis* grown in soil were air-dried for 0, 1, 2, and 4 h. Each value represents the mean of three independent measurements. Bars indicate the standard deviation of the mean. Different letters denote statistically significant differences at *P* < 0.05 (Tukey’s test), following a one-way ANOVA test with treatment as the variable factor. **(B)** Quantitative RT-PCR analysis of wax biosynthetic genes in 4-week-old *Arabidopsis* wild type (Col-0) and *RAP2.4* OX lines. The RNAs were extracted from rosette leaves of *Arabidopsis* grown in soil for three weeks. Each value represents the mean of three independent measurements. Bars indicate the standard deviation of the mean. Different letters denote statistically significant differences at *P* < 0.05 (Tukey’s test), following a one-way ANOVA test with treatment as the variable factor.

Next, we examined whether the significant decrease in total wax loads and levels of alkanes observed in *rap2.4-1* and *rap2.4-2* leaves relative to that in the wild type under drought stress conditions correlates with the reduced expression of genes involved in cuticular wax biosynthesis and its regulation. Total RNA was isolated from wild type and *rap2.4* mutant leaves, which were air-dried for 0, 1, 2, and 4 h and then subjected to qRT-PCR analysis. The *RAP2.4* gene displayed the highest expression in wild type leaves 1 to 2 h after drought treatment. The expression of *RD29A* (*At5g52310*), which is known as a drought-inducible gene (Shinozaki and Yamaguchi-Shinozaki, 2007), was upregulated in both wild type and *rap2.4* mutant leaves 1 h after drought treatment and continuously increased in the wild type up to 4 h after drought treatment. However, no further increase in the levels of *RD29A* was detected in *rap2.4* mutant leaves 2 and 4 h after drought treatment. Increased levels of *KCS2* (*At1g04220*) and *CER1* (*At1g02205*) transcripts were approximately 20% and 40% lower, respectively, in *rap2.4* leaves than in wild type leaves 4 h after drought treatment (Figure 6A). However, no significant differences were observed in the levels of *KCR1* (*At1g67730*), *PAS2* (*At5g10480*), *KCS6* (*At1g68530*), *CER3* (*At5g57800*), *MYB94* (*At3g47600*), and *MYB96* (*At5g62470*) expression between wild type and *rap2.4* leaves after drought treatment (Supplementary Figure 3).

In addition, the expression of *KCS2* and *CER1* was markedly upregulated in all three *RAP2.4* OX lines relative to that in the wild type (Figure 6B). The levels of *KCS1* (*At1g01120*), *KCS6*, *CER3*, and *CER4*/*FAR3* (*At4g33790*) were increased by 1.2- to 2.0-fold in 2 or 3 *RAP2.4* OX lines compared with that in the wild type, but no significant change in the levels of *PAS2* and *ECR* (*At3g55360*) expression was observed between the wild type and *RAP2.4* OX lines (Figure 6B). These results indicate that *KCS2* and *CER1* are directly or indirectly regulated by the *RAP2.4* transcription factor under drought.

### Transcriptional Activation Assay of RAP2.4 in *N. benthamiana* Protoplasts

**FIGURE 7:**
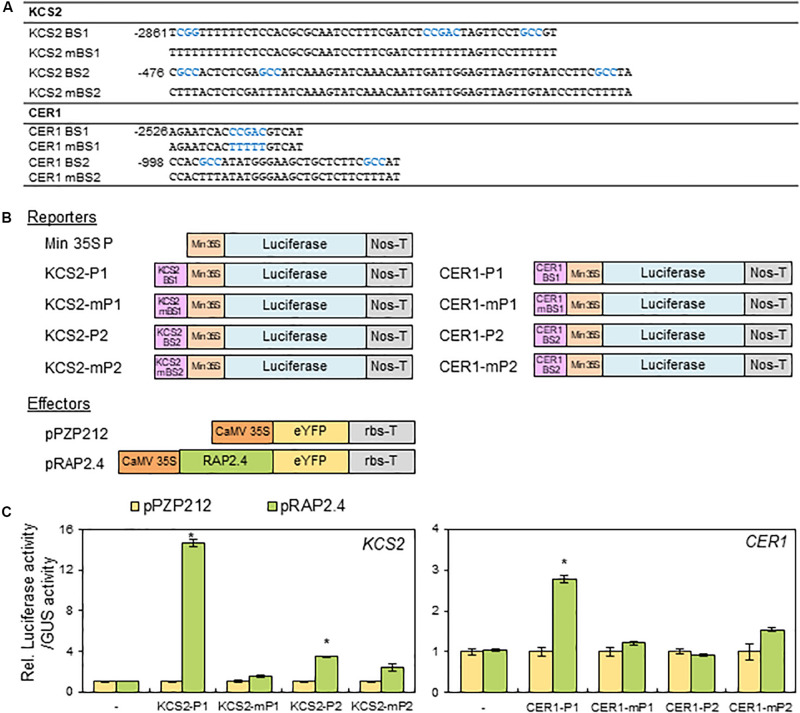
Transactivation assay of *RAP2.4* in *N. benthamiana*. **(A)** The oligonucleotide sequences (BS) including RAP2.4 binding consensus sequences, which are present in the promoter regions of *KCS2* and *CER1* genes. Core binding sequences shown in blue were mutated, resulting in mBSs, to verify specific binding. **(B)** Schematic diagrams of reporter and effector constructs for transcriptional activation assay. For the reporter constructs, the synthesized oligonucleotides were annealed, and then the DNA fragments were ligated between the CaMV 35S minimal promoter (Min 35S) and the luciferase gene. For the effector construct, *RAP2.4* was translationally fused with the gene encoding eYFP between the CaMV 35S promoter (CaMV35SP) and the terminator of ribulose 1,5-biphosphate carboxylase/oxygenase small subunit from *Pisum sativum* (rbs-T). Nos-T, The terminator of the nopaline synthase gene. **(C)** Transcriptional activation assay in *N. benthamiana* leaf protoplasts. *N. benthamiana* leaf protoplasts were co-transformed with reporter and effector constructs, and luciferase and GUS activities were determined fluorometrically. Luciferase activity was normalized by dividing this value by GUS activity. Each value is the mean of four independent measurements (*t* test, *P < 0.01). Bars indicate the standard error of the mean.

Based on the observation that the expression of *KCS2* and *CER1* was altered in *rap2.4* mutants and *RAP2.4* OX lines compared with that in the wild type, we further examined whether RAP2.4 directly regulate the expression of *KCS2* and *CER1*. According to the previous report that RAP2.4 specifically recognizes the CCGAC core sequences of the drought response element (DRE) and GCC box (Rae et al., 2011), the consensus CCGAC and GCC motifs were searched in the promoter regions of *KCS2* and *CER1*. For reporter constructs, the selected DNA fragments (KCS2 BS1, KCS2 BS2, CER1 BS1, and CER1 BS2] containing the consensus CCGAC or GCC motifs and their mutated DNA fragments (KCS2 mBS1, KCS2 mBS2, CER1 mBS1, and CER1 mBS2), where the consensus CCGAC and GCC sequences were converted to TTTTT and TTT sequences, respectively, were transcriptionally ligated to the minimal *CaMV 35S* promoter in the pBI:LUC harboring the luciferase reporter gene (Figures 7A,B). For effector constructs, the coding region of *RAP2.4* was inserted between the *CaMV 35S* promoter and nopaline synthase (Nos) terminator in the *pPZP212* vector and named *pRAP2.4* (Figure 7B). The reporter and effector vectors were co-transfected into *N. benthamiana* protoplasts with a vector harboring *GUS* for internal control to monitor transformation efficiencies and luciferase and GUS activities were measured. The ratio of LUC activity to GUS activity in protoplasts expressing *LUC* driven by the *KCS2-P1, KCS2-P2*, and *CER1-P1* promoters was approximately 14.5-, 3.5-, and 2.8-fold elevated, respectively, but no significant increase in *LUC* gene expression driven by the *CER1-P2* promoter was observed, upon co-expression with *RAP2.4* compared with the *pPZP212* control. By contrast, the co-transformation of *pRAP2.4* with *KCS2-mP1, KCS2-mP2, CER1-mP1, or CER1-mP2* did not elevate the *LUC* gene expression (Figure 7C), indicating that the CCGAC or GCC consensus motifs present in the *KCS2* (BS1 and BS2) and *CER1* (BS1) promoter regions are essential for the expression of *KCS2* and *CER1* by RAP2.4 and RAP2.4 may be directly involved in the transcriptional activation of *KCS2* and *CER1* genes.

## Discussion

When land plants face water deficit stress, they close the stomata (Brodribb and McAdam, 2017) and the aerial organs of land plants increase total loads of cuticular waxes, which are present in the outermost layer of the cuticle, to reduce non-stomatal water loss (Riederer and Schreiber, 2001; Domínguez et al., 2017). In particular, an increase in the levels of alkanes among the diverse wax components is prominent in *Arabidopsis* under drought (Kosma et al., 2009; Lee and Suh, 2015a). Although involvement of MYB96 and MYB94 transcription factors has been reported in the upregulation of cuticular wax biosynthesis in *Arabidopsis* (Seo et al., 2011; Lee and Suh, 2015b; Lee et al., 2016), increased total wax loads were still observed in *myb96 myb94* mutants relative to the wild type under drought. This observation prompted us to find another transcription factor, which upregulates cuticular wax biosynthesis in *Arabidopsis* under drought. In this study, we show that ABA-, drought-, osmotic-, and salt-stress inducible *RAP2.4* activates cuticular wax biosynthesis in *Arabidopsis* leaves under drought stress conditions. qRT-PCR and transcriptional activation assays revealed that RAP2.4 upregulates the expression of *KCS2* and *CER1*, which are required for the production of alkanes, and contributes to an increase in total wax loads which is critical for drought resistance in *Arabidopsis* leaves.

The aerial parts of land plants increase or decrease total wax loads by the up- or down-regulation of genes involved in cuticular wax biosynthesis under different environmental conditions such as drought, dark, or pathogen infection or in an organ-specific manner (Raffaele et al., 2008; Buschhaus and Jetter, 2011; Seo et al., 2011; Go et al., 2014; Park et al., 2016). Therefore, it has been speculated that cuticular wax biosynthesis is mainly regulated by transcriptional regulatory mechanism, which is supported by the identification of several transcription factors, WIN1/SHN1, MYB30, MYB96, MYB94, DEWAX, DEWAX2, and WRI4 involved in the regulation of wax biosynthesis (Aharoni et al., 2004; Raffaele et al., 2008; Seo et al., 2011; Go et al., 2014; Lee and Suh, 2015b; Park et al., 2016; Kim et al., 2018). Among the several transcription factors, ABA- and drought-inducible MYB96 and MYB94 transcription factors were reported to additively function in the upregulation of cuticular wax biosynthesis under both well-watered and water-deficit conditions (Lee et al., 2016). However, the wax-deficient phenotype was observed in the leaves of *rap2.4-1* and *rap2.4-2* mutants under only drought stress conditions (Figure 3), suggesting that *RAP2.4* plays a role in the upregulation of cuticular wax biosynthesis under drought. The hypothesis is consistent with the enhanced expression of *RAP2.4* by ABA, salt, drought, and osmotic stress treatments (Figure 1B). In addition, in microarray analyses of 2-week-old Arabidopsis WT versus *myb96-D* (*MYB96* OX) leaves and 3- to 4-week-old Arabidopsis WT versus *MYB94* OX leaves, the levels of *RAP2.4* expression was only approximately 1.4- and 1.2-fold elevated in *myb96-D* (*MYB96* OX) and *MYB94* OX leaves relative to WT, respectively (Seo et al., 2011; Lee et al., 2016). No significant differences were observed in the levels of *MYB94* and *MYB96* expression between Arabidopsis WT and *rap2.4* leaves 1 h and 2 h after drought treatment (Supplementary Figure 2). Therefore, these results suggest that a DERB-type RAP2.4 transcription factor and MYB96/MYB94 transcription factors might independently regulate cuticular wax biosynthesis under drought stress conditions.

In wax chemical analysis in the leaves of *rap2.4-1* and *rap2.4-2* relative to that of the wild type under drought, a significant reduction was observed in the levels of alkanes, C29, C31, and C33, which comprise approximately 85% of total wax loads (Figure 3). Whereas a remarkable increase in the alkanes loads was observed in the leaves of *RAP2.4* OX lines, although an elevation in the content of other components was detected (Figure 4). These results are consistent with the upregulation of *KCS2* and *CER1*, which function in VLCFA and alkane synthesis, respectively (Figure 6). Transcriptional activation assay (Figure 7) showed that the expression *KCS2* and *CER1* is increased by the expression of *RAP2.4* in tobacco protoplasts, indicating that *KCS2* and *CER1* are the target genes of RAP2.4. Among wax biosynthetic genes, the expression of *KCS2* and *CER1* genes are the most highly induced (approximately 10- and 5-fold, respectively) in *Arabidopsis* in response to drought (Seo et al., 2011). In *myb96 myb94* seedlings, approximately 2- or 10-fold increase in the levels of *KCS2* and *CER1* transcripts were still detected under drought-treated conditions compared with that in the well-watered conditions (Lee et al., 2016). In addition to MYB96 and MYB94, therefore, RAP2.4 functions in the upregulation of *KCS2* and *CER1* under drought. In particular, electrophoretic mobility shift assay (EMSA) and/or chromatin immunoprecipitation (ChIP) assay showed that MYB96 and MYB94 transcription factors directly interact with the same cis-element (TAACTACTAACTA) in the promoter region of *KCS2* gene, suggesting that it is possible that ABA- and drought-induced expression of *KCS2* is synergistically regulated by MYB96 and MYB94 transcription factors (Seo et al., 2011; Lee et al., 2016). However, neither MYB96 nor MYB94 transcription factors were able to specifically bind to the putative consensus sequences in the *CER1* promoter region (Seo et al., 2011; Lee et al., 2016). In this study, transcriptional activation assay of RAP2.4 in *N. benthamiana* protoplasts revealed that the CCGAC or GCC consensus motifs in the promoter regions of *KCS2* and *CER1* are required for the expression of *KCS2* and *CER1* driven by RAP2.4 (Figure 7), indicating that ABA- and drought-inducible RAP2.4 may be directly involved in the transcriptional activation of *KCS2* and *CER1* genes under drought.

The stability of the MYB96 and MYB30 proteins was reported to be controlled by MYB30-INTERACTING E3 LIGASE 1 (MIEL1) E3 ubiquitin ligase, which controls protein turnover (Lee et al., 2017). In particular, Lee et al. (2017) reported that wax accumulation is balanced in *Arabidopsis* stems by preventing the adverse effects of excess MYB96 protein through degradation of MYB96. Based on the evidence that the transcript levels of *MIEL1* decreased significantly in 18-day-old *Arabidopsis* under osmotic (300 mM mannitol), salt (150 mM NaCl), and drought stress conditions^3^, MYB96 could be stabilized to play a role in the activation of wax biosynthesis under drought (Seo et al., 2011). It was reported that BTB/POZ and MATH domain (BPM) proteins, which are substrate adaptors for Cullin 3-based E3 ubiquitin ligase, are negative regulators to control the stability of AP2/ERF transcription factors, DREB2A and WRI1, which are critical in the modulation of heat stress response and fatty acid metabolism, respectively (Chen et al., 2013; Morimoto et al., 2017). As Weber and Hellmann (2009) reported that RAP2.4 interacts with BPM proteins, it will be interesting to investigate whether the BPM and RAP2.4 interaction is involved in the molecular mechanisms underlying the regulation of drought-induced wax biosynthesis. The recent evidence that the Kelch-domain containing F-box protein, Small and Glossy Leaves 1(SAGL1) regulates cuticular wax biosynthesis by modulating the stability of CER3 in response to changes in humidity (Kim et al., 2019) supports that post-translational regulatory mechanism as well as transcriptional and post-transcriptional control mechanisms play important roles in the regulation of cuticular wax biosynthesis (Aharoni et al., 2004; Hooker et al., 2007; Raffaele et al., 2008; Seo et al., 2011; Lam et al., 2012, 2015; Go et al., 2014; Lee and Suh, 2015b; Park et al., 2016; Kim et al., 2018, 2019).

RAP2.4/WOUND INDUCED DEDIFFERENTIATION 1 (WIND1) has been reported to be implicated in plant development regulated by light and ethylene (Lin et al., 2008) and promotion of somatic cell dedifferentiation in response to wounding (Iwase et al., 2011). In particular, Lin et al. (2008) reported that *RAP2.4* OX seedlings or plants displayed shorter hypocotyls, reduced apical hook curvature in darkness, increased and longer root hairs, reduced size of cotyledons, and early flowering. In addition to the developmental defects in *RAP2.4* OX plants, overexpression of *RAP2.4* confers an enhanced tolerance to drought stress (Lin et al., 2008), but little was known about the molecular mechanism underlying drought tolerance in *RAP2.4* OX plants. In this study, we observed a wax-deficient phenotype in drought-treated *rap2.4-1* and *rap2.4-2* mutants, even though no noticeable phenotype was observed in the *rap2.4* mutants (Lin et al., 2008). In conclusion, an AP2/DREB-type transcription factor, RAP2.4, is a transcriptional activator which upregulates *KCS2* and *CER1* genes and thereby, the total wax loads increased in *Arabidopsis* leaves under drought. This study also suggests that RAP2.4 might be useful in the production of alkanes and the development of crops with enhanced drought tolerance.

## Data Availability Statement

All datasets for this study are included in the article/Supplementary Material.

## Author Contributions

SY, HK, and MS conceived and designed the research and wrote the manuscript. SY, HK, and RK conducted the experiments. SY, HK, RK, JK, and MS analyzed the data. JK edited the manuscript. All authors read and approved the manuscript.

## Conflict of Interest

The authors declare that the research was conducted in the absence of any commercial or financial relationships that could be construed as a potential conflict of interest.

## Footnotes

1 https://www.arabidopsis.org/

2 https://www.novoprolabs.com/vector/V10826, pRAP2.4.

3 http://bar.utoronto.ca/efp/cgi-bin/efpWeb.cgi
