# Supplementary material for: AP2/DREB Transcription Factor RAP2.4 Activates Cuticular Wax Biosynthesis in Arabidopsis Leaves Under Drought
Source: Front Plant Sci. 2020 Jul 3;11:895. doi: 10.3389/fpls.2020.00895 (PMC7347990; doi:10.3389/fpls.2020.00895)
Supplement: Supplementary file 1 [file Presentation_1.pdf]

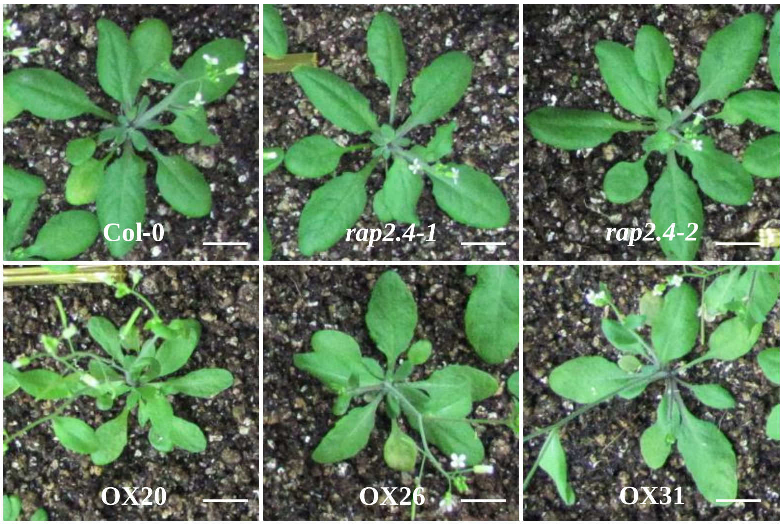

Supplementary Figure 1. Phenotypes of 3- to 4week-old wild type (Col-0), *rap2.4* mutants, *rap2.4-1* and *rap2.4-2*, and *RAP2.4* overexpression lines (OX20, OX26, and OX31). Bars = 1 cm.

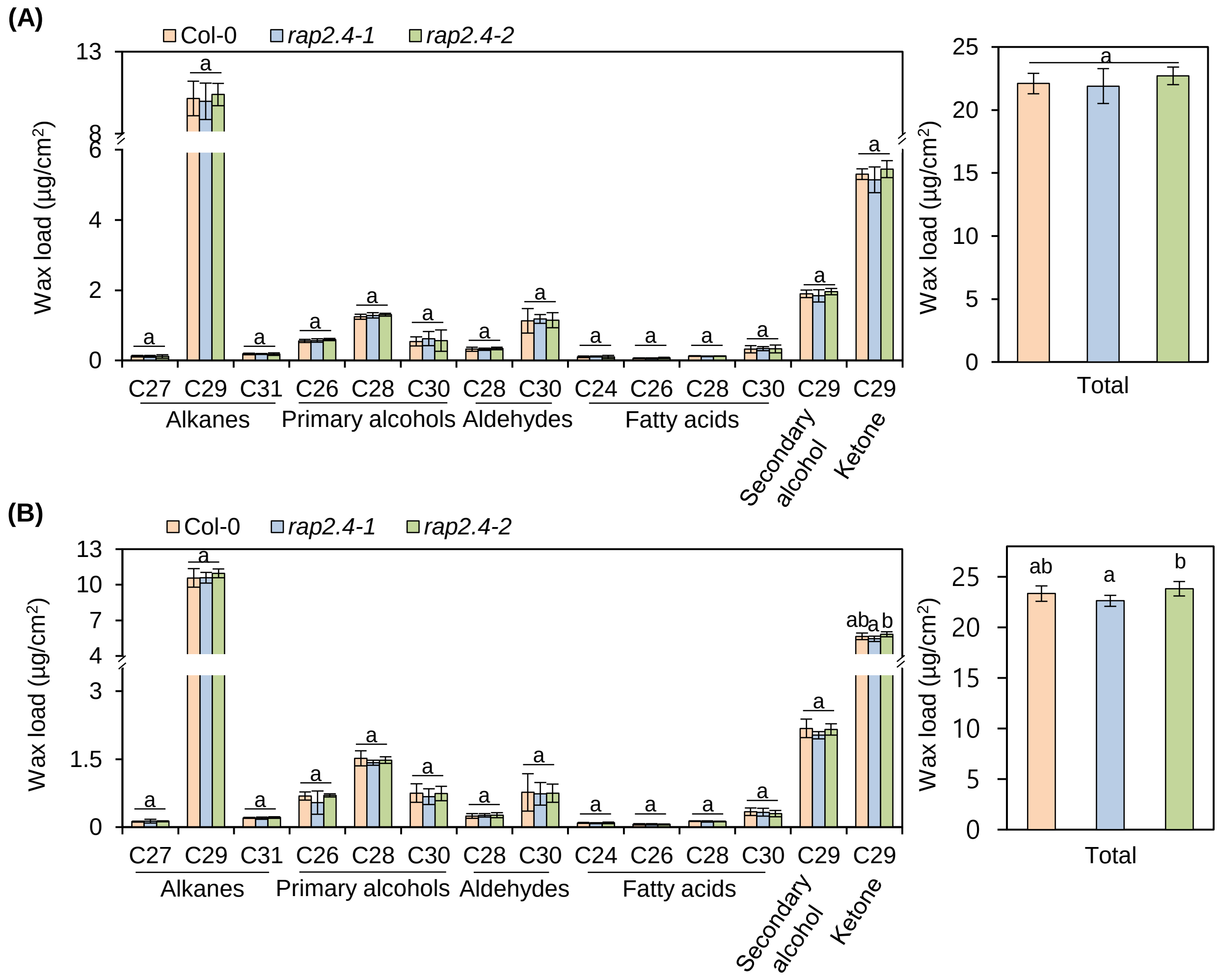

Supplementary Figure 2. Cuticular wax composition and amount in the stems of wild type (Col-0) and *rap2.4* mutants under the normal and drought conditions.

(A) Cuticular wax composition and amount in stems of plants grown under normal growth conditions (temperature, 24 ± 2 ℃; humidity, approximately 50%; light condition, 100-120 μmol $m^{-2}s^{-1}$).

(B) Cuticular wax composition and amount in stems of plants grown under drought stress conditions.

Cuticular waxes were extracted from stems of 5- to 6-week-old Arabidopsis plants grown under normal growth or drought stress conditions. Each value is the mean of three independent measurements. Bars indicate standard deviation. Different letters denote statistically significant differences at $P < 0.05$ (Tukey's test), following a one-way ANOVA test with treatment as the variable factor.

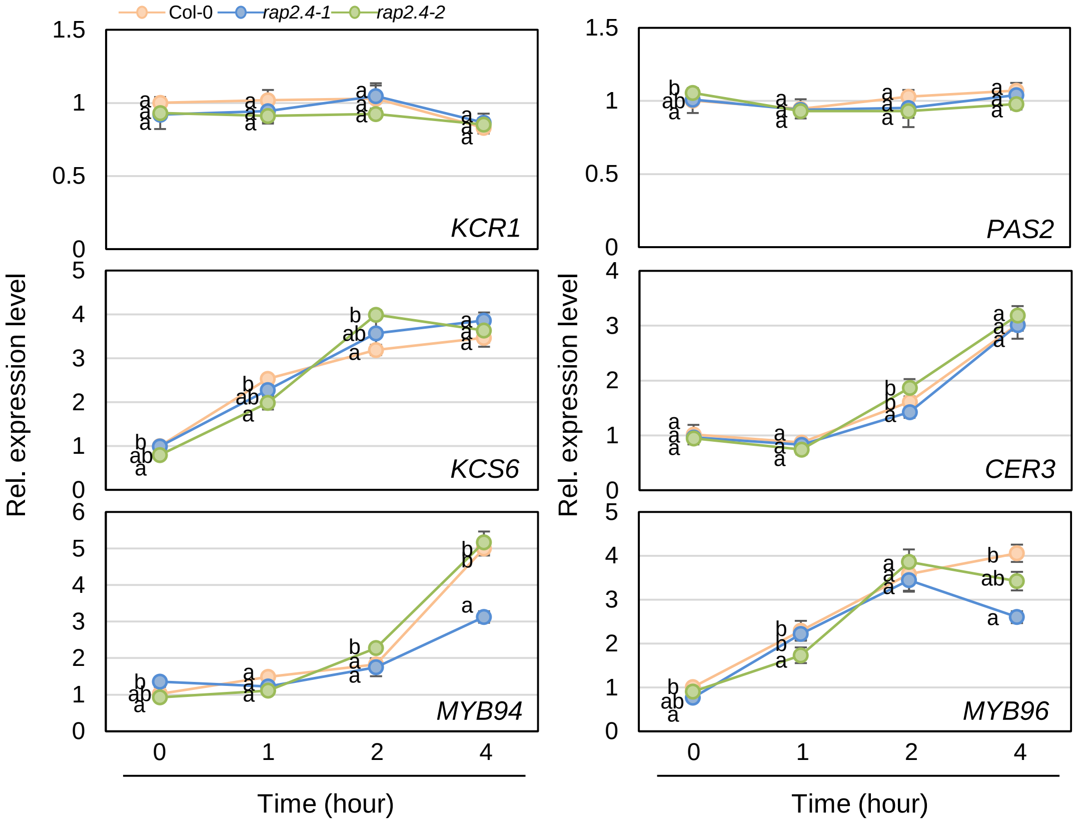

Supplementary Figure 3. Quantitative RT-PCR analysis of wax biosynthetic genes in 4-week-old (Col-0) and *rap2.4* mutants.

The transcript level of *KCR1*, *PAS2*, *KCS6* and *MYB96* in 4-week-old wild type (Col-0) and *rap2.4* mutants. Leaves from 4-week-old Arabidopsis plants grown in soil were air-dried for 0, 1, 2, and 4 h. Each value represents the mean of three independent measurements. Bars indicate the standard deviation of the mean. Different letters denote statistically significant differences at $P < 0.05$ (Tukey's test), following a one-way ANOVA test with treatment as the variable factor.
