# Supplementary material for: AP2/DREB Transcription Factor RAP2.4 Activates Cuticular Wax Biosynthesis in Arabidopsis Leaves Under Drought
Source: Front Plant Sci. 2020 Jul 3;11:895. doi: 10.3389/fpls.2020.00895 (PMC7347990; doi:10.3389/fpls.2020.00895)
Supplement: Supplementary file 2 [file Presentation_2.pdf]

Supplementary Table 1. Primers used in this study

Reaction	Primer name	Sequence information
RT-PCR analysis	At1g78080 F1	5'-GGAGCTCATGGCAGCTGCTATG
	At1g78080 R1	5'-CCCCGGGAGCTAGAATCGAATCC
qRT-PCR analysis	At1g78080-NF1	5'-CCATATGATGGCAGCTGCTATG
	At1g78080-NR1	5'-GCCCGGGAAGGAACACCTGAAC
Mutant isolation	At1g78080-NR1	5'-GCCCGGGAAGGAACACCTGAAC
	At1g78080-CF1	5'-CCATATGGTTCAGGTGTTCCTTC
	LBa1	5'-TGGTTCACGTAGTGGGCCATCG
Overexpression	At1g78080 F1	5'-GGAGCTCATGGCAGCTGCTATG
	At1g78080 R1	5'-CCCCGGGAGCTAGAATCGAATCC
qRT-PCR of wax biosynthetic genes	At1g13320 real F	5'-GCGGTTGTGGAGAACATGATACG
	At1g13320 real R	5'-GAACCAAACACAATTCGTTGCTG
	RD29A F1	5'-GATAACGTTGGAGGAAGAGTCGGC
	RD29A R1	5'-CAGCTCAGCTCCTGATTCACTACC
	KCS2 real F2	5'-CAACCTCGCTTTCCAACAAA
	KCS2 real R2	5'-TCCGGTTTTCTCAAGCACTG
	CER1 real F3	5'-AGGTCGACAGGGAGACCAAC
	CER1 real R3	5'-ATAAGCGCTGCCATCAACAC
	CER3 real F2	5'-GGAAACGCAACGTTATTGGA
	CER3 real R2	5'-AGCGTAACCGTAGATCGCAC
	CER4 real F2	5'-ACCGTGGACCAACAAAGAAG
	CER4 real R2	5'-GCAATCAAGTAGCGTATGGTCA
	MYB94 real F3	5'-GGAAACTGGAGATCTGTGCCTACT
	MYB94 RT R2	5'-CTTGAAGCATAGACCCCAGATG
	MYB96 real F1	5'-GGAAACTGGAGATCTGTCCCAACA
	MYB96 real R1	5'-GATGAAGAGACACCATCATTATCTTC
	PAS2 real F2	5'-GAGCTCTCTCCAAATCCAAGAGG

	PAS2 real R2	5'-GTTCAAGTAATCGCCGAGGAAGTA
	KCR1 real-F1	5'-GGCTTATACCGAAGCTAAGG
	KCR1 real-R1	5'-CCGGTTCAACCAATCATTGC
	ECR real F1	5'-TCAACATCGCTACTCAGACC
	ECR real R2	5'-GGAATGGAGGAAGTATCACCCATC
	KCS1 real F2	5'-GTAAGCACGGAAAACATAACCCTA
	KCS1 real R2	5'-CGTCTGATCCTTTATGTGTTCGA
	KCS6 real F1	5'-GTGAAGCCCTCAAGGCAAAC
	KCS6 real R1	5'-CGAAGGCCAGCTTGAAATCC
	KCS20 real F	5'-CAGATGCTTCAGGTGCAACC
	KCS20 real R	5'-TGAACGGCTGCGATCAGA
Transactivation assay	Min35S pro_*Pst*I F	5'-GCCCTGCAGTCGACCGCAAGACCCTTCCTCTATATAAGG
	Min35S pro *Xba*I R	5'-GTCTCTAGACTAGTTGTAATTGTAAATAGTAATTGTAATGTTGTTTGTTG
	Luc_*Xma*I F	5'-GAACCCGGGATGGAAGACGCCAAAAACATAAAGAAAGG
	Luc_*Sac*I R	5'-GCGGAGCTCTTACACGGCGATCTTTCCGC
	CER1 pro1 *Hin*dIII F1	5'-AGCTTAATTTGACCGACTCACTATG
	mCER1 pro1 *Hin*dIIIF1	5'-AGCTTAATTTGATTTTTTCACTATG
	CER1 pro1 *Sal*I R1	5'-TCGACATAGTGAGTCGGTCAAATTA
	mCER1 pro1 *Sal*I R1	5'-TCGACATAGTGATTTTTTCAAATTA
	CER1 pro2 *Hin*dIII F1	5'-AGCTTAGAATCACCCGACGTCATG
	mCER1 pro2 *Hin*dIII F1	5'-AGCTTAGAATCACTTTTTGTCATG
	CER1 pro2 *Sal*IR1	5'-TCGACATGACGTCGGGTGATTCTA
	mCER1 pro2 *Sal*I R1	5'-TCGACATGACGTCTTTTTATTCTA
	KCS2 pro1 *Hin*dIII F1	5′-AGCTTTCGGTTTTTTCTCCACGCGCAATCCTTTCGATCTCCGACTAGTTCCTGCCGTCG
	mKCS2 pro1 *Hin*dIII F1	5′-AGCTTTTTTTTTTTTCTCCACGCGCAATCCTTTCGATCTTTTTTTAGTTCCTTTTTTCG
	KCS2 pro1 *Sal*I R1	5′-TCGACGACGGCAGGAACTAGTCGGAGATCGAAAGGATTGCGCGTGGAGAAAAAACCGAA
	mKCS2 pro1 *Sal*I R1	5′-TCGACGAAAAAAGGAACTAAAAAAAGATCGAAAGGATTGCGCGTGGAGAAAAAAAAAAA
	KCS2 pro2 *Hin*dIII F1	5′-AGCTTCGCCACTCTCGAGCCATCAAAGTATCAAACAATTGATTGGAGTTAGTTGTATCCTTCGCCTACG
	mKCS2 pro2 *Hin*dIII F1	5′-AGCTTCTTTACTCTCGATTTATCAAAGTATCAAACAATTGATTGGAGTTAGTTGTATCCTTCTTTTACG
	KCS2 pro2 *Sal*I R1	5′-TCGACGTAGGCGAAGGATACAACTAACTCCAATCAATTGTTTGATACTTTGATGGCTCGAGAGTGGCGA
	mKCS2 pro2 *Sal*I R1	5′-TCGACGTAAAAGAAGGATACAACTAACTCCAATCAATTGTTTGATACTTTGATAAATCGAGAGTAAAGA
